# Supplementary figures and images for: Contraction-induced endocardial id2b plays a dual role in regulating myocardial contractility and valve formation
Source: eLife. 2025 Jul 24;13:RP101151. doi: 10.7554/eLife.101151 (PMC12289310; doi:10.7554/eLife.101151)

IB: anti-Flag

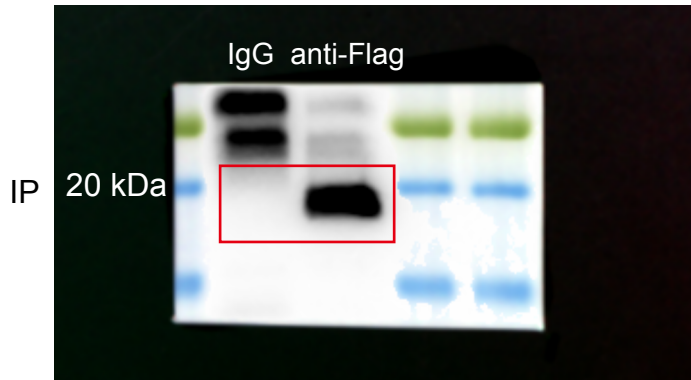

IB: anti-HA

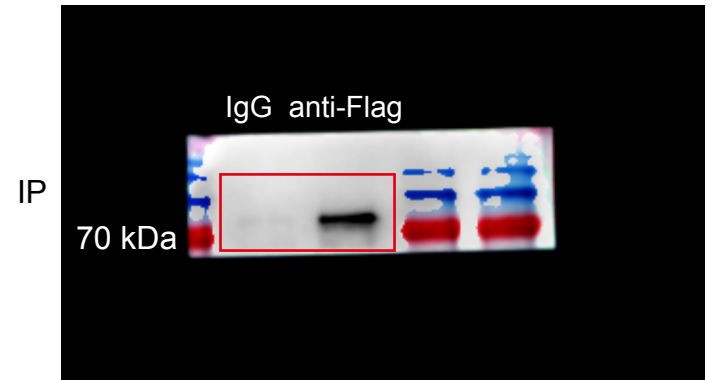

IB: anti-Flag

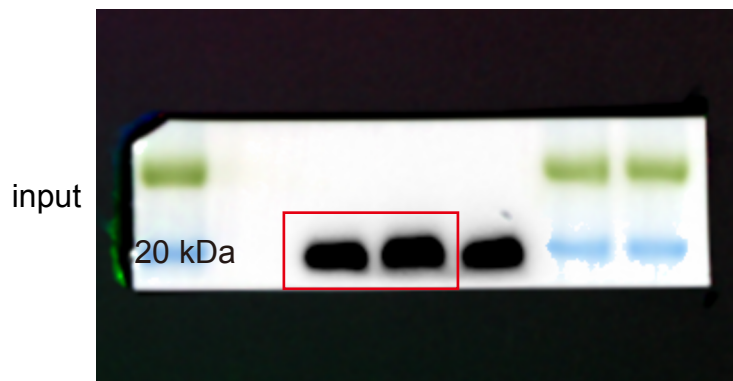

IB: anti-HA

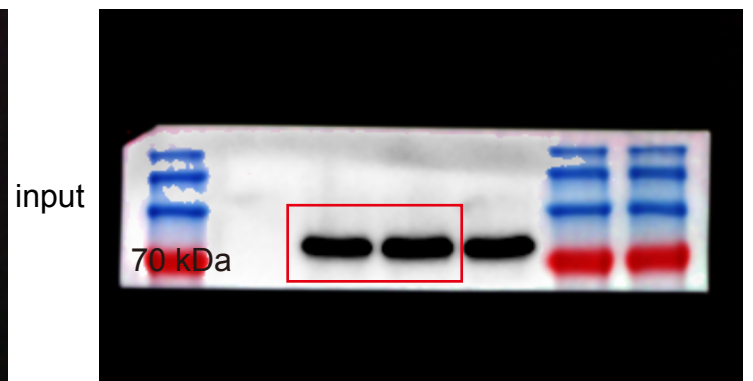

Supplement: Figure 7—source data 1. [file elife-101151-fig7-data1.zip › source data 1.pdf]

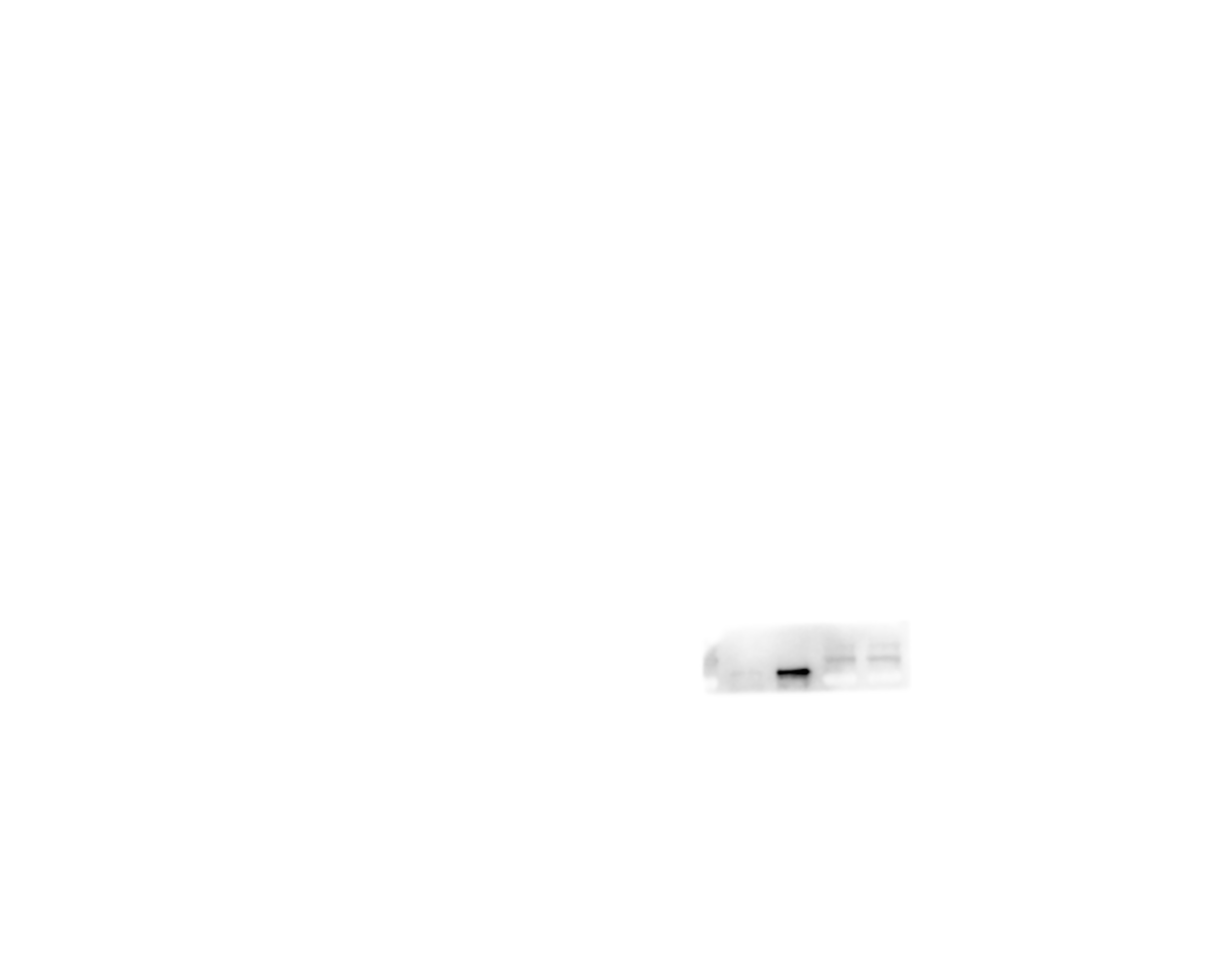

Supplement: Figure 7—source data 2. [file elife-101151-fig7-data2.zip › source data 2/Original tiff shown in Figure 7A-IP-anti-HA.tif]

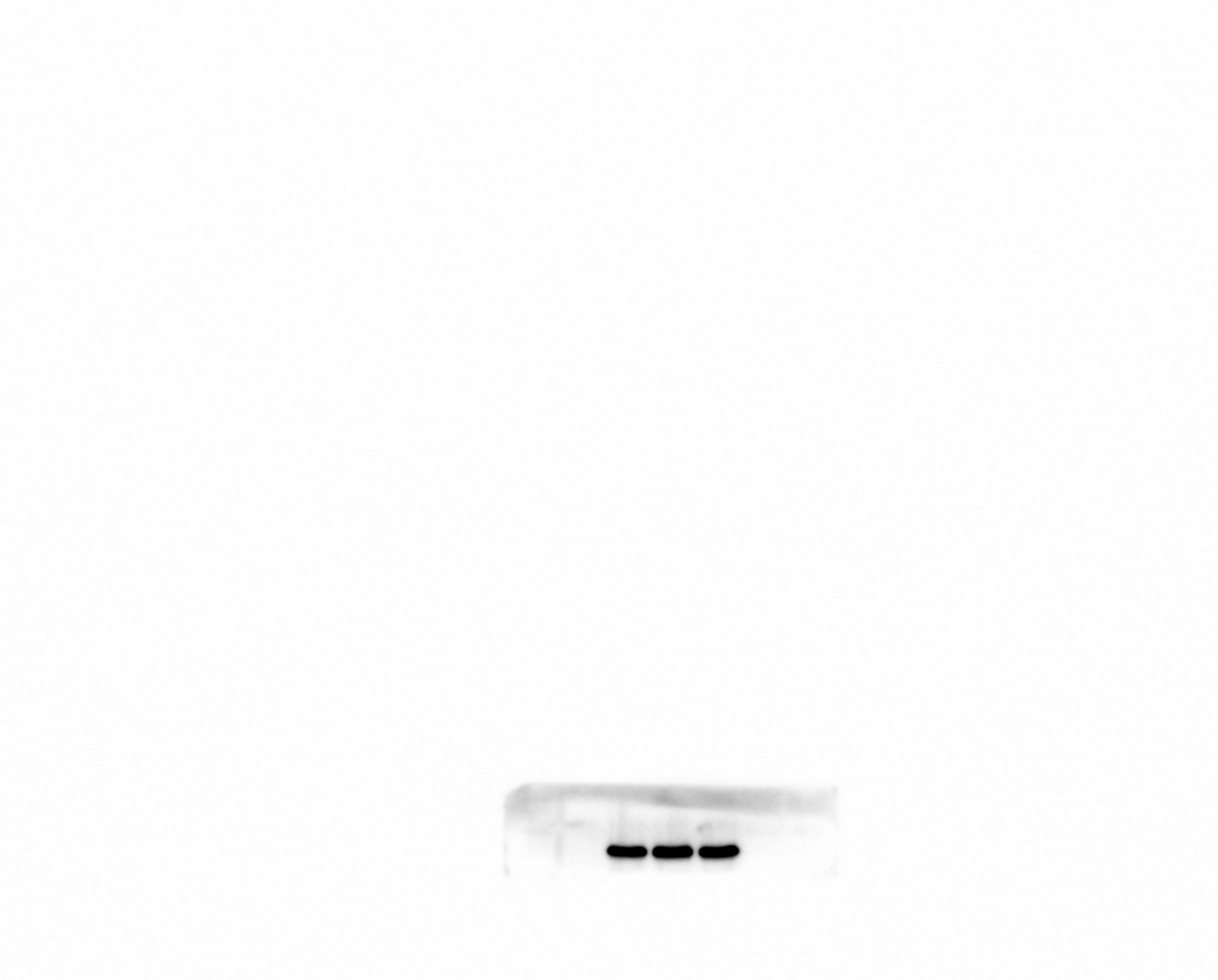

Supplement: Figure 7—source data 2. [file elife-101151-fig7-data2.zip › source data 2/Original tiff shown in Figure 7A-input-anti-HA.tif]

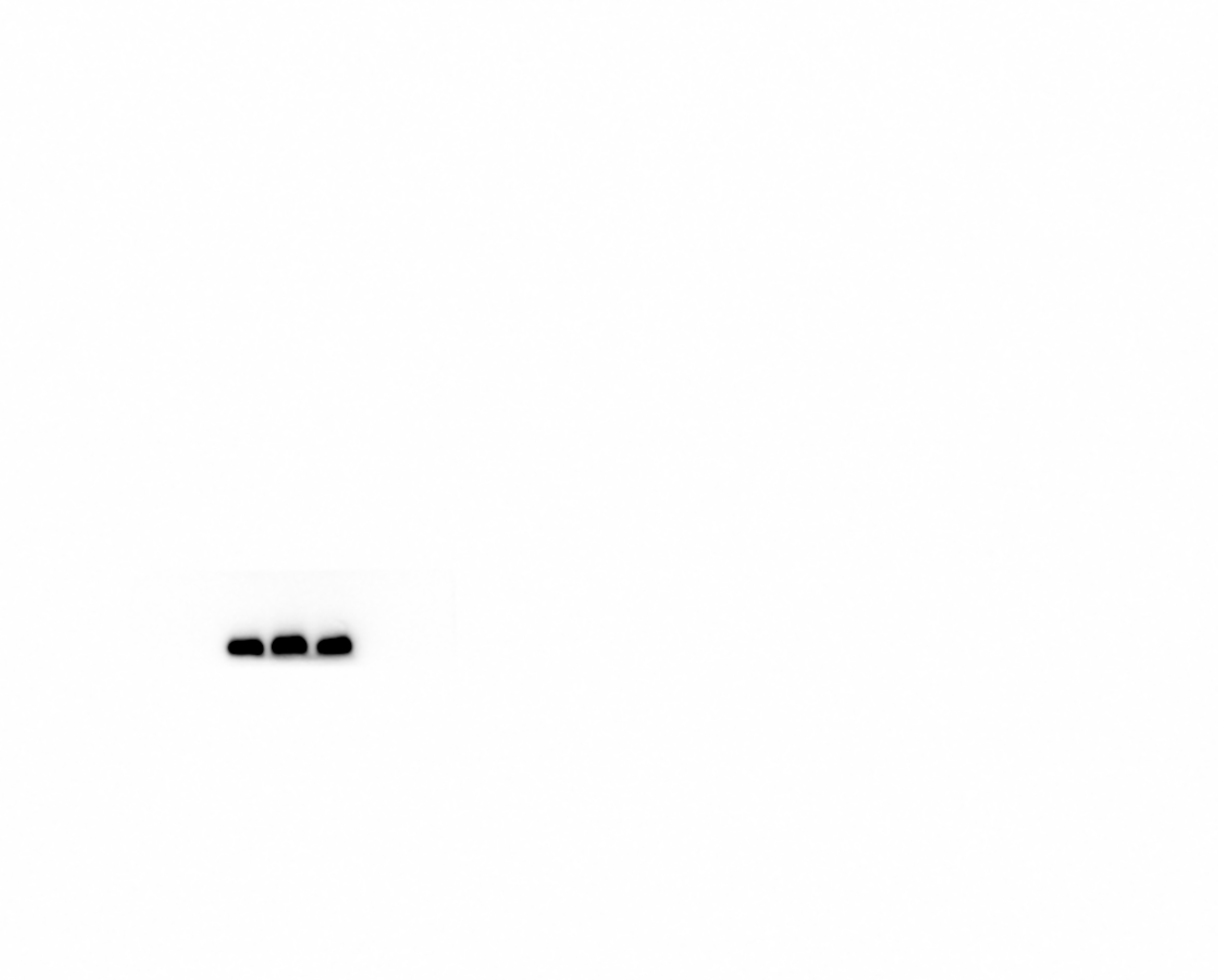

Supplement: Figure 7—source data 2. [file elife-101151-fig7-data2.zip › source data 2/Original tiff shown in Figure 7A-input-anti-Flag.tif]

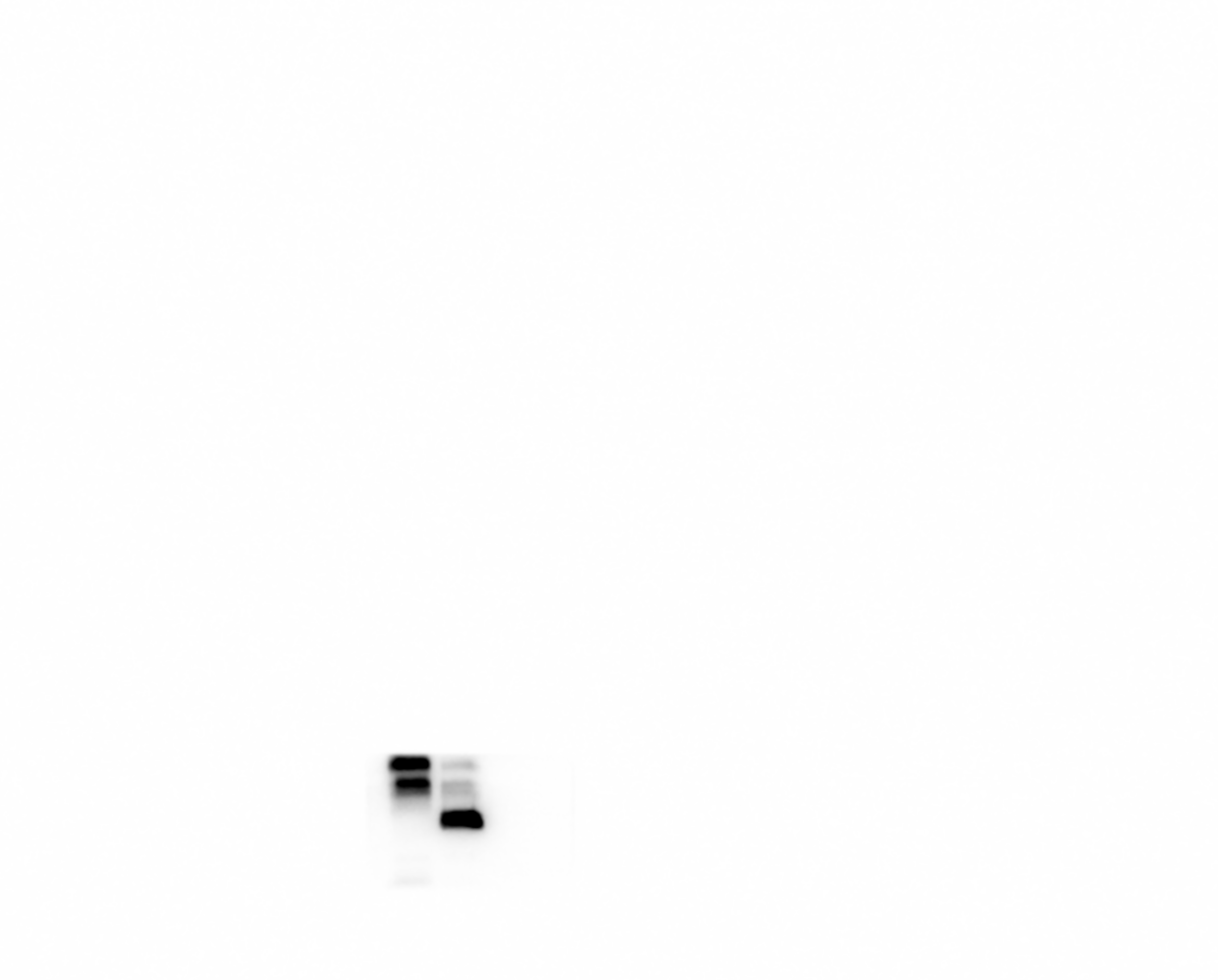

Supplement: Figure 7—source data 2. [file elife-101151-fig7-data2.zip › source data 2/Original tiff shown in Figure 7A-IP-anti-Flag.tif]
